# Anti-tumour activity of longikaurin A (LK-A), a novel natural diterpenoid, in nasopharyngeal carcinoma

**DOI:** 10.1186/1479-5876-11-200

**Published:** 2013-08-28

**Authors:** Qing-Feng Zou, Ji-Ke Du, Hua Zhang, Hong-Bo Wang, Ze-Dong Hu, Shu-Peng Chen, Yong Du, Man-Zhi Li, Dan Xie, Juan Zou, Han-Dong Sun, Jian-Xin Pu, Mu-Sheng Zeng

**Affiliations:** 1 Section 3 of Internal Medicine, The Affiliated Tumor Hospital of Guangzhou Medical University, Guangzhou, Guangdong, People’s Republic of China; 2 State Key Laboratory of Oncology in South China, Department of Experimental Research, Sun Yat-sen University Cancer Center, Guangzhou, Guangdong, People’s Republic of China; 3 Kunming Institute of Botany, Chinese Academy of Science, Kunming, Yunnan, People’s Republic of China; 4 Department of Head and Neck Surgery, The Third Affiliated Hospital of Kunming Medical University, Kunming, People’s Republic of China

**Keywords:** Longikaurin A (LK-A), Apoptosis, Caspase, Nasopharyngeal carcinoma

## Abstract

**Background:**

Longikaurin A is a natural *ent*-kaurene diterpenoid isolated from *Isodon* genus. The *ent*-kaurene diterpenoids isolated from medicinal plants have been shown to have anti-disease effects. The present study was designed to examine the anti-tumour effects of longikaurin A (LK-A) in nasopharyngeal carcinoma *in vitro* and *in vivo.*

**Methods:**

Apoptosis and cell cycle arrest were determined by flow cytometry analysis of the cells treated with Longikaurin A. The proteins of apoptosis signaling pathway were detected by western blotting analysis. Finally, we examined whether LK-A exhibits anti-tumour activity in xenograft models.

**Results:**

Longikaurin A inhibited the cell growth by inducing apoptosis and cell cycle arrest. At low concentrations, longikaurin A induced S phase arrest and at higher concentrations, longikaurin A induced caspase-dependent apoptosis by regulating apoptotic molecules. Finally, longikaurin A significantly inhibited the tumour growth of CNE2 xenografts *in vivo* and showed no obvious effect on the body weights of the mice.

**Conclusion:**

Our results suggest that Longikaurin A exhibited anti-tumour activity in nasopharyngeal carcinoma *in vitro* and *in vivo.*

## Background

Nasopharyngeal carcinoma (NPC) is one of the most common malignant tumours in China. Although the clinical cure rate of early NPC is very high, the mortality rate of nasopharyngeal carcinoma accounts for 2.82% of all malignant cancer-related deaths in China [1]. Patients with advanced nasopharyngeal carcinoma have a poor prognosis and high mortality even after treatment. Moreover, multi-drug resistance is a difficult problem faced in the treatment of advanced or intracranially-recurrent nasopharyngeal carcinomas. Thus, there is an urgent need to develop a more effective therapeutic agent. Medicinal plants have been used for thousands of years to treat a variety of diseases [2,3]. In recent decades, extracts from herbal medicines have been investigated for the treatment of many malignant tumors, and plants have been a source for new anti-cancer drugs. For example, vinblastine was traditionally obtained from *Catharanthus roseus*, taxol was isolated from the bark of the Pacific yew tree *Taxus brevifolia*, and camptothecin was isolated from the bark and stem of *Camptotheca acuminata*[4-6].

**Figure 1:**
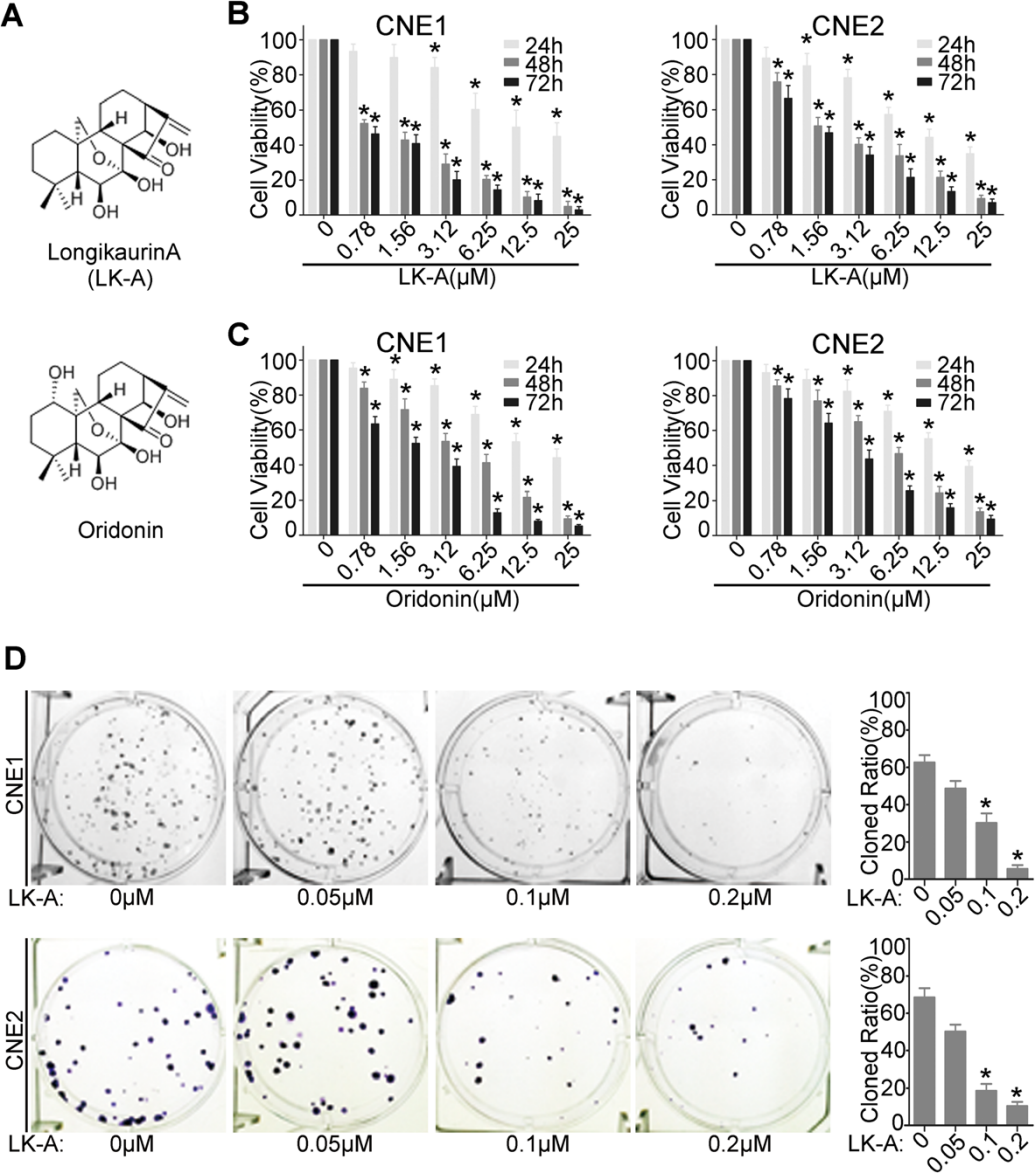
**LK-A inhibits cell viability and colony formation of the human NPC cells CNE1 and CNE2. (A)** The chemical structural of LK-A and oridonin. **(B, C)** A comparison of the cytotoxic effects of LK-A and oridonin on NPC cells. CNE1 and CNE2 (0, 0.78, 1.56, 3.12, 6.25, 12.5 and 25 μΜ) of LK-A or oridonin for 24, 48 and 72 hrs. Cell viability was determined by MTT assays. The IC_50_ values 48 hrs after treatment were 1.26 ± 0.17 μM, 1.52 ± 0.22 μM, 3.66 ± 0.37 μM and 5.93 ± 0.48 μM for **B** and **C** , respectively. Data are shown as the mean ± SD of three independent experiments. *p < 0.05 vs. control group (untreated cells). **(D)** The colony forming ability of NPC cells was inhibited by LK-A treatment. Bar chart showing the decreased proportion of the cloned ratio after treatment with LK-A. Data are shown as the mean ± SD of two independent experiments. *p < 0.05 vs. control group (untreated cells).

Oridonin (Figure 1A) is a natural *ent*-kaurene diterpenoid extracted from *Isodon* genus that has attracted much attention because of its anti-tumour activity [7,8]. Oridonin has been safely used for the treatment of hepatoma and promyelocytic leukaemia in China for many years. Longikaurin A (LK-A) (Figure 1A) is a natural *ent*-kaurene diterpenoid isolated from *Isodon* genus too. LK-A is structurally similar to oridonin. It has been recently reported that LK-A induces apoptosis in multiple myeloma H929 cells [9]. However, it is unknown whether LK-A exerts anti-tumour effects in solid tumours. In this study, we examine the effects of LK-A on nasopharyngeal carcinoma through *in vitro* and *in vivo* experiments.

## Materials and methods

### Chemicals and antibodies

LK-A was obtained from the leaves of *Isodon ternifolius* (D. Don) Kudô, which were collected in Jinxiu, Guangxi, China. The dried and milled plant material (10 kg) was extracted four times by incubation with 100 L of 70% aqueous Me_2_CO for 3 days at room temperature and was subsequently filtered. The filtrate was evaporated under reduced pressure and then partitioned with EtOAc (4 × 60 L). The EtOAc partition (938.5 g) was applied to a silica gel (200–300 mesh), and six fractions, A-F, were eluted with CHCl_3_‒Me_2_CO (1:0–0:1). Fraction B (618.5 g) was decolorised on an MCI gel and eluted with 90% MeOH-H_2_O to yield fractions B1-B4. Fractions B1 (116 g) and B2 (135 g) were further separated by repeated silica gel column chromatography to isolate LK-A (20 g). The LK-A powder was dissolved in dimethyl sulphoxide (DMSO) at a concentration of 50 mM and stored at −20°C. The working concentrations used in this study were freshly diluted in medium before each experiment. The DMSO concentration was kept below 0.1% when used in cell culture and did not exert any detectable effect on cell growth or death. Cell culture reagents including RPMI 1640 medium and keratinocyte/serum-free medium were purchased from Invitrogen (Carlsbad, USA). The following monoclonal antibodies were used for western blotting: Bax (1:1000, Cell Signaling, Massachusetts, USA), BCL-X_L_ (1:2000, Cell Signaling, Massachusetts, USA), Akt and phospho-Akt (1:1000, Cell Signaling, Massachusetts, USA), Phospho-GSK-3β (1:1000, Millipore, Bedford, USA), and α-tubulin (1:3000, Santa Cruz Biotechnology, Santa Cruz, CA). Annexin V and PI (Invitrogen, Carlsbad, USA) were also used for flow cytometry. All other chemicals including BSA, Coctail, PBS and Tween-20 were purchased from Sigma.

### Cell culture

The well-differentiated nasopharyngeal carcinoma cell line (CNE1) and the poorly differentiated nasopharyngeal carcinoma cell line (CNE2) were maintained in our laboratory. The two cell lines were cultured in RPMI1640 medium supplemented with 5% foetal bovine serum (Invitrogen). Immortalised nasopharyngeal epithelial cells (NPEC2) induced by Bmi-1 were established as described previously and grown in keratinocyte/serum-free medium (Invitrogen) [10]. All cell lines were incubated at 37°C in a 5% CO_2_, 95% humidified atmosphere.

### MTT cell viability assay

In total, 2.5 × 10^3^ cells were seeded into 96-well plates, incubated overnight and then treated with various concentrations of LK-A dissolved in DMSO for 24, 48 and 72 hrs. Subsequently, 10 μl of 3-(4,5-dimethylthiazol-2-yl)-2,5-diphenyltetrazolium bromide (MTT, 5 mg/ml) was added to each well, and the plate was incubated at 37°C for 4 hrs. The supernatant was then carefully removed, and 150 μL/well dimethyl sulfoxide (DMSO) was added to dissolve the formazan crystals. The absorbance of the solubilised product was measured with a microplate spectrophotometer at 490 nm (μQuant, Biotek, USA). This experiment was performed in quadruplicate and repeated 3 times. Using the formula below, we calculated the percent cell viability for each concentration of LK-A (data are shown as the mean values ± SD). The IC_50_ was determined with SPSS 17.0.

### Colony formation assay

First, 300 CNE1 and 200 CNE2 cells were plated per well in six-well plates. After an overnight incubation, the cells were treated with various concentrations of LK-A dissolved in DMSO. As negative control, some cells were treated with vehicle (DMSO) only. One week later, the cells were fixed with methanol for 15 min and then stained with 0.1% crystal violet for 15 min. After washing away the crystal violet, the plates were photographed. To objectively quantify the colonies, Quantity One software was used to count colonies that were larger than the average parameter and had a minimum signal intensity of 1.0 or greater. At least two independent experiments were performed for each assay.

### Apoptosis assay

In total, 1.5 × 10^5^ cells per well were seeded into 6-well plates and incubated overnight. Then, cells were treated with various concentrations of LK-A for 48 hrs. Briefly, the cells were then harvested, washed in PBS, and incubated with Alexa-488 and propidium iodide for cellular staining in binding buffer at room temperature for 15 min in the dark. Stained cells were immediately examined by flow cytometry on a FC500 cytometer (Beckman Coulter). For experiments in which the pan-caspase inhibitor Z-VAD-FMK was used, it was added 2 hrs before the addition of LK-A.

### Western blotting analysis

CNE1 and CNE2 cells were seeded into 6-well plates and incubated overnight. The cells were then treated with various concentrations of LK-A for 48 hrs. Western blotting analysis was performed as previously described [10]. Where relevant, the blots were probed with the antibodies indicated in the figures, and the signals were detected with an enhanced chemiluminescence (ECL) reagent (Amersham Pharmacia Biotech, Piscataway, NJ). The membranes were stripped and probed with an anti-alpha-tubulin mouse monoclonal antibody (Santa Cruz Biotechnology, Santa Cruz, CA) to confirm equal loading of the samples.

### Cell cycle analysis

First, 1 × 10^5^ cells were seeded into 6-well plates and incubated overnight. Cells were then treated with various concentrations of LK-A for 36 hrs. The cells were harvested, washed with cold PBS and then fixed for 12 hrs with 70% ethanol in PBS at 4°C. Subsequently, the cells were resuspended in PBS containing 100 μg/ml RNase and 50 μg/ml PI and incubated at 37°C for 30 min. Cell cycle distribution of nuclear DNA was determined by flow cytometry on a FC500 cytometer (Beckman Coulter).

### Tumour formation assay

Nude mice were purchased from Sun Yat-sen University Experimental Animal Center. They were cared in accordance with the institution guidelines. 4 × 10^5^ CNE2 cells were suspended in 100 μl of RPMI 1640 medium with 25% Matrigel (BD Biosciences) and inoculated subcutaneously into the right flanks of 5-week-old nude mice. Six days later, the tumours were approximately 5 mm × 5 mm. The nude mice were randomly divided into four groups based on tumour size. Mice were injected intraperitoneally with either the vehicle (saline) every other day, LK-A (6 mg/Kg) once a week, LK-A (6 mg/Kg) every other day, or the positive control drug Paclitaxel (Sigma; 30 mg/Kg) once a week for three weeks. The mice were monitored every other day for palpable tumour formation, and the tumours were measured using a Vernier calliper. We calculated tumour volume using the following formula: 4π/3 × (width/2)^2^ × (length/2) [11]. Three weeks later, we stopped the injections and continued to observe the mice for another week. After this period, the mice were sacrificed, and the tumours were removed for analysis.

## Results

### LK-A inhibits cell viability and colony formation of the human NPC cells CNE1, CNE2

To determine whether LK-A exhibits anti-tumour effects against NPC, we treated the NPC cell lines CNE1 and CNE2 with various concentrations of LK-A. An MTT assay was used to analyse the growth rates of the cell lines at 24 hrs, 48 hrs and 72 hrs. Compared with the vehicle, LK-A inhibited CNE1 and CNE2 cell growth in a time- and dose-dependent manner (Figure 1B). While there were not substantial differences at 24 hrs, at 48 and 72 hrs after treatment, the cell viability was significantly decreased, even at LK-A concentrations of less than 1 μM. The IC_50_ values at 48 hrs of treatment were 1.26 ± 0.17 μM and 1.52 ± 0.22 μM for CNE1 and CNE2 cells, respectively. Thus, these data suggest that LK-A has a substantial dose- and time-dependent cytotoxic effect on NPC cells.

Previous studies have shown that oridonin exerted significant cytotoxic effects on many types of malignant tumour cell lines, such as the human leukaemia cell line HL-60 [12], the human hepatoma cell line HepG2 [13] and the human melanoma cell line A357-S2 [14]. Furthermore, oridonin inhibits CNE1 and CNE2 cell growth in a time- and dose-dependent manner. The IC_50_ values at 48 hrs of treatment were 3.66 ± 0.37 μM and 5.93 ± 0.48 μM for CNE1 and CNE2 cells, respectively (Figure 1C). Thus, these data suggest that the cytotoxic effect of LK-A on NPC cell lines is substantially stronger than that of oridonin. However, they have a similar cytotoxic effec on immortalised nasopharyngeal epithelial cells (NPEC2-Bmi-1). The IC_50_ values at 48 hrs after treatment with LK-A and oridonin were 2.96 ± 0.32 μM and 3.15 ± 0.48 μM NPEC2-Bmi-1 cells, respectively (Additional file 1: Figure S1A). The differences between LK-A and oridonin in cytotoxic effect on NPC cells and NPEC2-Bmi-1 cell were intuitively showed in Additional file 1: Figure S1B. We next determined the long-term effects of LK-A by performing a colony formation assay. We found that the cells treated with LK-A formed fewer and smaller colonies in a dose-dependent manner compared with control-treated cells (Figure 1D). The concentrations of LK-A used in this assay were well below the IC_50_ values determined in the MTT assay. Still, these low concentrations could inhibit NPC cell growth for long periods of time.

### LK-A induces apoptosis in NPC cells

**Figure 2:**
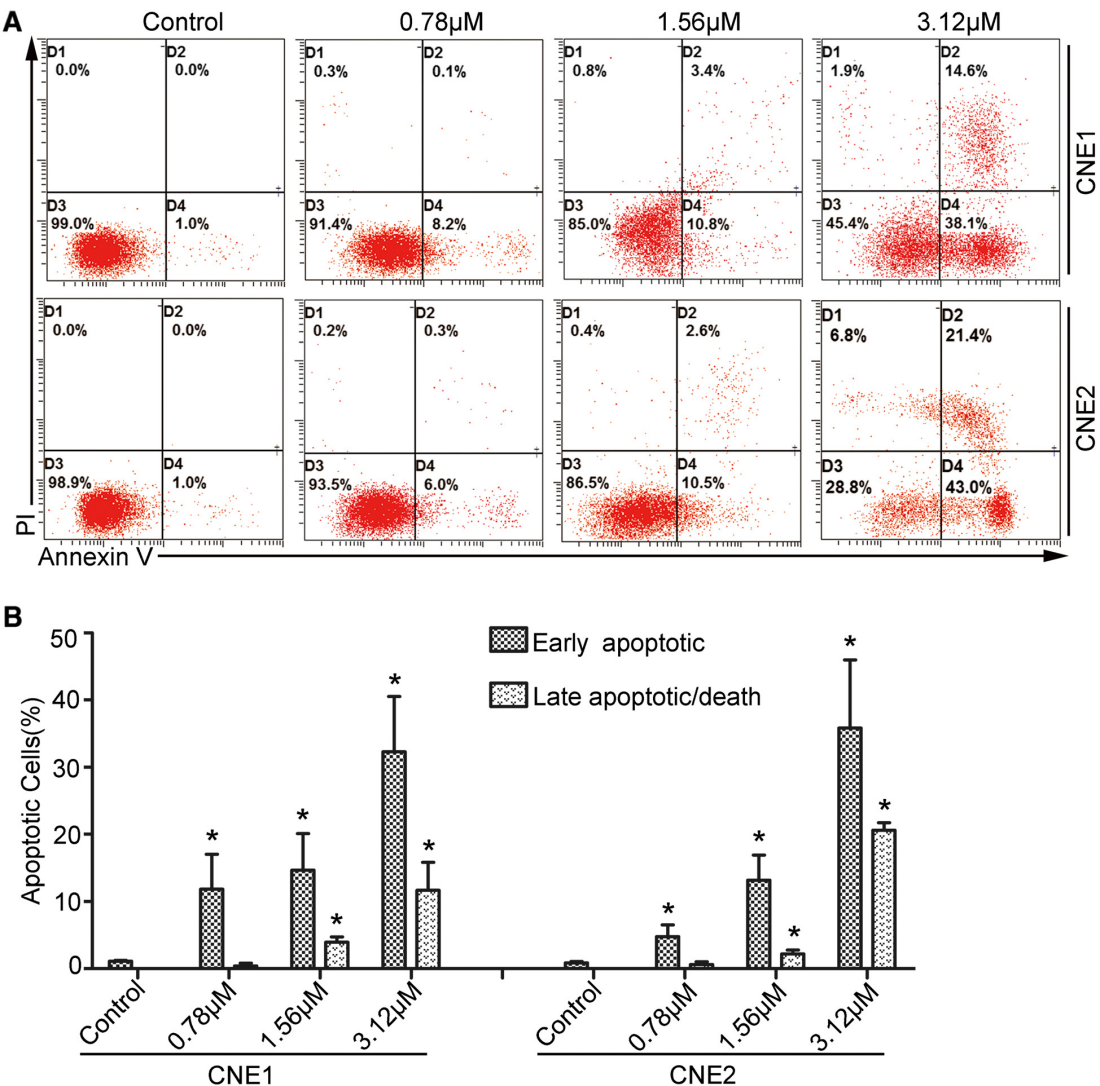
**LK-A induces apoptosis of human nasopharyngeal carcinoma cells.** Flow cytometry analysis of CNE1 and CNE2 cells treated with 0.78, 1.56 and 3.12 μΜ LK-A for 48 hrs. **(A)** Dot plots showing the percentage of viable (D3), early apoptotic (D4), late apoptotic (D2) and necrotic (D1) cells. **(B)** Bar chart indicating the increased proportion of early and late apoptotic cells after treatment with LK-A. Data are shown as the mean ± SD from two independent experiments. *p < 0.05 vs. control group (untreated).

Given that LK-A has been shown to induce apoptosis in multiple myeloma H929 cells [9], we examined whether LK-A could induce apoptosis in NPC cells. Vehicle-treated or LK-A-treated CNE1 and CNE2 cells were stained with Annexin V and PI. Flow cytometry analysis of the cells identified four groups: viable cells (Annexin V- PI-), early apoptotic cells (Annexin V + PI-), late apoptotic cells (Annexin V + PI+) and necrotic cells (Annexin V- PI+) cells. As shown in Figure 2A and B, treatment with three different concentrations of LK-A (0.78, 1.56 and 3.12 μM) resulted in increased amounts of apoptotic cells in a dose-dependent manner (11.85 ± 5.16%, 14.65 ± 5.44%, 32.3 ± 8.21% in CNE1 cells; 4.75 ± 1.76%, 13.15 ± 3.75%, 35.5 ± 3.18% in CNE2 cells, respectively); however, only 1% of the vehicle-treated cells were apoptotic. A dose-dependent increase in late apoptotic cells was also observed (0.4 ± 0.33%, 3.95 ± 0.77%, 11.7 ± 4.1% in CNE1 cells and 0.6 ± 0.42%, 2.2 ± 0.56%, 20.8 ± 1.13% in CNE2 cells) compared to untreated cells (0%). However, LK-A exerted a similar effect on immortalised nasopharyngeal epithelial cells (NPEC2-Bmi-1). Additional file 2: Figure S2.

### LK-A up-regulates cleaved caspases 3 and 9 and PARP

**Figure 3:**
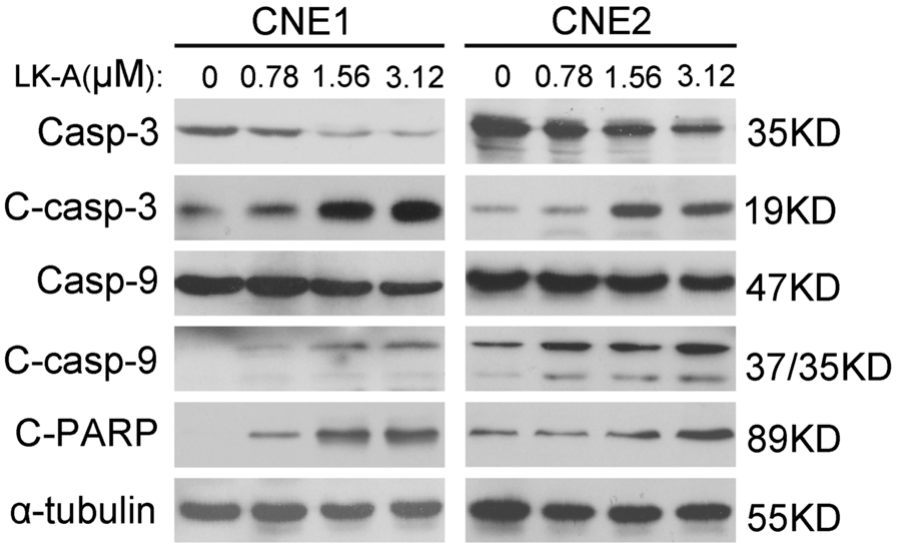
**LK-A induces apoptosis through the intrinsic caspase pathway.** Expression levels of cleaved caspase-3, -9 and cleaved PARP were elevated in a dose-dependent manner in CNE1 and CNE2 cells after treated with LK-A for 48 hrs. α-tubulin served as a loading control.

Apoptosis is a complex process. The caspase-dependent pathway plays a vital role in the apoptotic process, which can be further divided into the extrinsic or intrinsic pathways [15-17]. Both the intrinsic and extrinsic pathways involve activation of caspases 3 and 7 that cleave a broad spectrum of cellular target proteins, including poly(ADP-ribose) polymerase and cause cell death. Therefore, we performed a Western blot analysis of LK-A-treated NPC cells. As shown in Figure 3, we observed a gradual increase in cleaved caspase 9, 3 and cleaved PARP in both CNE1 and CNE2 cells treated with LK-A at different concentrations (0.78, 1.56 and 3.12 μM) compared to vehicle-treated cells. In contrast, we observed a gradual decrease in pro-caspase 9 and pro-caspase 3. Thus, our data suggested that LK-A could induce the activation of the intrinsic caspase pathway in both NPC cell lines.

**Figure 4:**
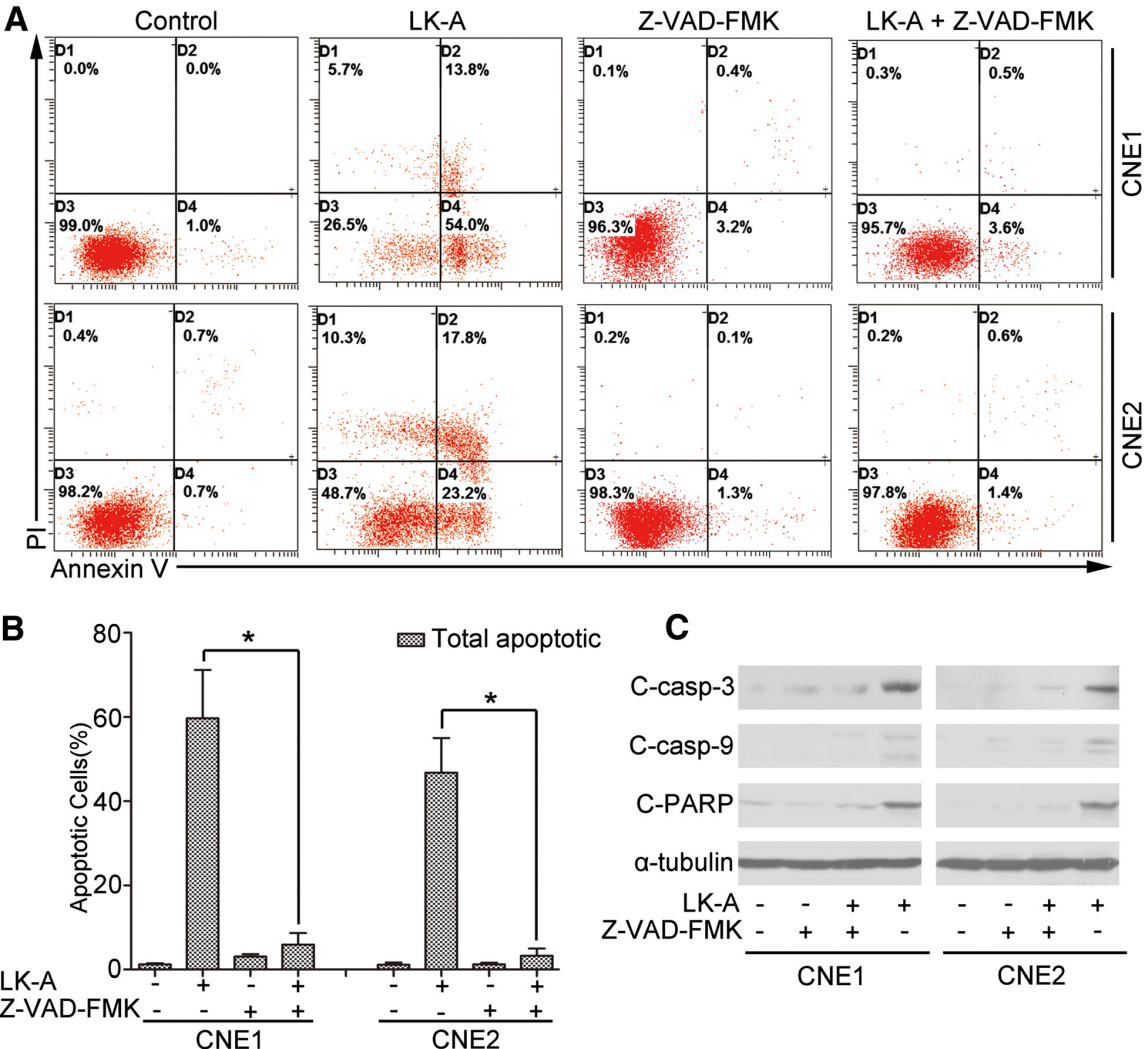
**Z-VAD-FMK blocks LK-A-induced apoptosis. (A)** Flow cytometry analysis of CNE1 and CNE2 cells treated with LK-A (3.12 μΜ) with or without Z-VAD-FMK for 48 hrs. **(B)** Bar chart showing that the proportion of total apoptotic cells decreased significantly after treatment with LK-A plus Z-VAD-FMK compared to LK-A alone. Data are shown as the mean ± SD from two independent experiments (*p < 0.05). **(C)** Western blotting analysis of CNE1 and CNE2 cells treated with LK-A (3.12 μΜ) with or without Z-VAD-FMK (20 μΜ) for 48 hrs.

We next examined whether the activation of caspase is required for the LK-A-mediated induction of apoptosis. We used the pan-caspase inhibitor Z-VAD-FMK [18], which specifically blocks the caspase-dependent cell apoptotic pathway. As shown in Figure 4, treatment of both NPC cells with LK-A and the pan-caspase inhibitor resulted in an obvious decrease in the amount of early and late apoptotic cells. Then, we performed a Western blot analysis of LK-A plus Z-VAD-FMK treated NPC cells. As shown in Figure 4C, we observed the expression level of cleaved caspase 3, 9 and cleaved PARP were significantly decrease in both CNE1 and CNE2 cells after treated by LK-A plus Z-VAD-FMK compared treated by LK-A only. Thus, caspase activation is required for LK-A-induced apoptosis in both NPC cell lines studied.

### LK-A regulates pro-apoptotic and anti-apoptotic molecules

**Figure 5:**
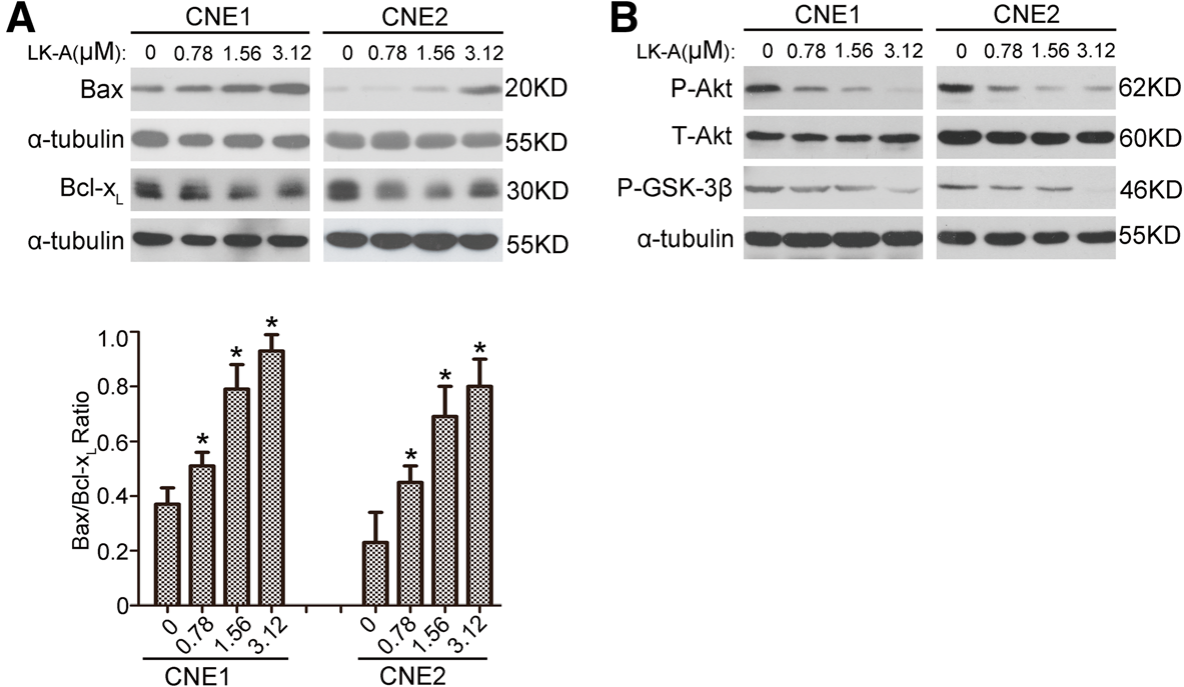
**LK-A regulates pro-apoptotic and anti-apoptotic molecules and inhibits PI3K/Akt pathway. (A)** Increased expression of the pro-apoptotic protein Bax and the ratio of Bax to Bcl-x_L_ occurred in a dose-dependent manner after treatment of CNE1 and CNE2 cells with LK-A for 24 hrs. *p < 0.05 vs. control group (untreated). **(B)** The phosphorylation levels of Akt and GSK-3 in CNE1 and CNE2 cells were downregulated after treatment with LK-A for 24 hrs.

The proteins of the Bcl-2 family play critical roles in the regulation of apoptosis by functioning as promoters (Bax) or inhibitors (Bcl-2 and Bcl-x_L_) of this cell death process [19-22]. To examine whether LK-A initiated apoptosis by affecting the cellular levels of pro-apoptotic and anti-apoptotic molecules, we performed Western blot assays. As shown in Figure 5A, our results indicated that LK-A treatment up-regulated the expression of the pro-apoptotic protein Bax and increased the ratio of Bax/Bcl-x_L_ in a dose-dependent manner.

### LK-A inhibits PI3K/Akt pathway

It has been shown previously that oridonin could inhibit the PI3K/Akt pathway to induce apoptosis in malignant tumour cells. Thus, we determined whether LK-A modulated the PI3K/Akt pathway in NPC cells. As shown in Figure 5B treatment of CNE1 and CNE2 cells with LK-A resulted in decreased levels of phosphorylated Akt and GSK-3β. These data indicate that LK-A down-regulates the phosphorylation levels of Akt and GSK-3β and thus modulates the PI3K/Akt pathway.

### LK-A induces S-phase cell cycle arrest in NPC cells

**Figure 6:**
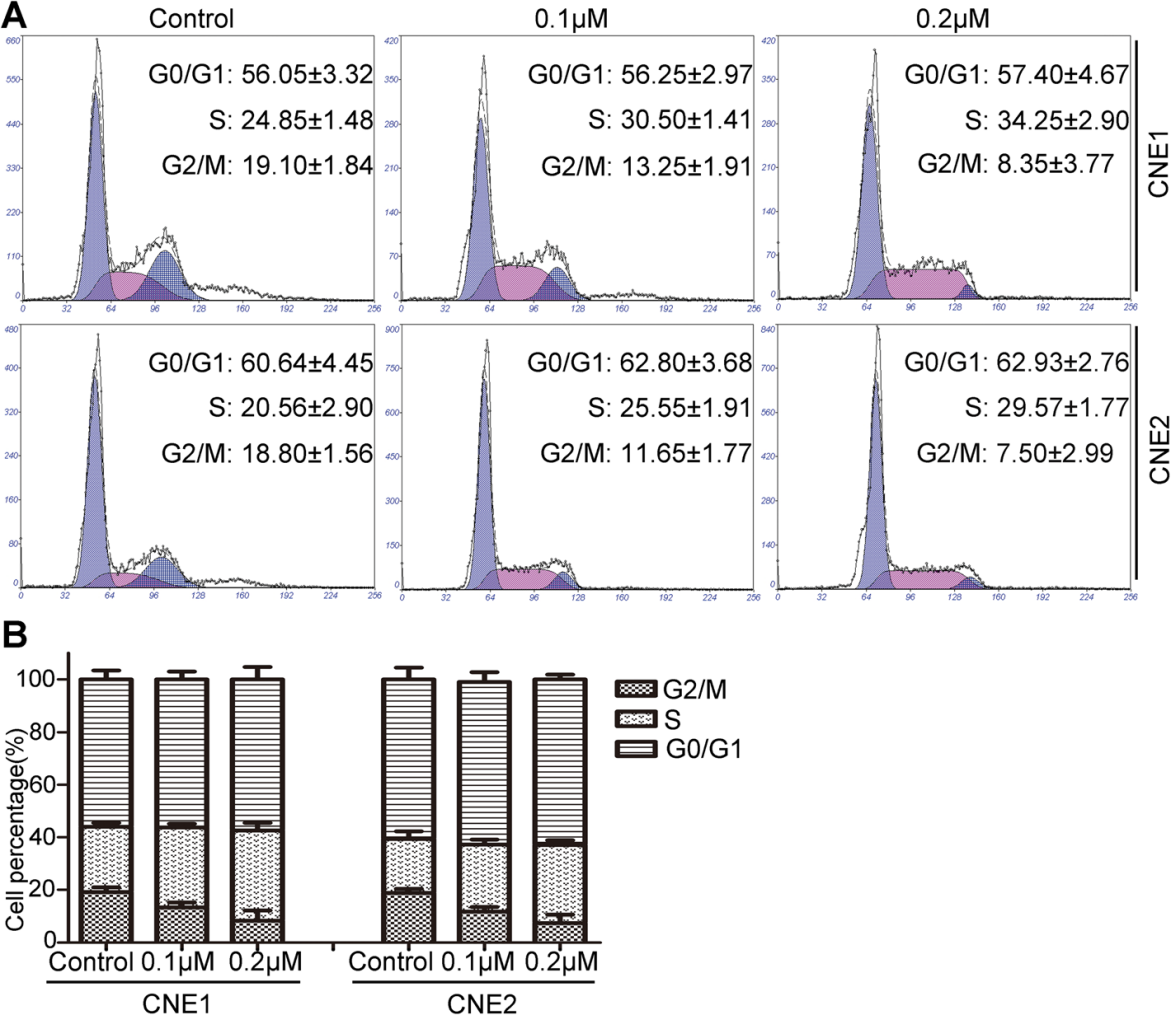
**Effect of LK-A on cell cycle progression of human nasopharyngeal carcinoma cells. (A)** Cell cycle distribution in CNE2 and CNE1 cells after treatment with different doses of LK-A. **(B)** Bar chart shows the accumulation of LK-A-treated cells in the S stage. Data are presented as the mean ± SD of two independent experiments.

Given that low concentrations of LK-A exerted a remarkable effect in the colony formation assay, we next examined whether LK-A affected the cell cycle distribution of NPC cells at doses lower than the IC_50_. As showed in Figure 6, CNE1 cells treated with 0.1 or 0.2 μM LK-A had more cells in S phase (30.50 ± 1.41% and 34.25 ± 2.90%, respectively) compared with vehicle-treated cells (24.85 ± 1.48%). Similar results were observed in CNE2 cells treated with 0.1 or 0.2 μM LK-A, with 25.55 ± 1.91% and 29.57 ± 1.77% of cells in S phase, respectively, compared with 20.56 ± 2.9% of untreated cells. In addition, LK-A treatment caused a concomitant decrease in the proportion of cells in G2/M phase of the cell cycle between untreated CNE1 cells (19.1 ± 1.84%) and treated CNE1 cells (13.25 ± 1.91% and 8.35 ± 3.77%, respectively) as well as between untreated CNE2 cells (18.8 ± 1.56%) and treated CNE2 cells (11.65 ± 1.77% and 7.5 ± 2.99%, respectively). Therefore, our data suggest that LK-A may induces cell cycle arrest at the S phase (Figure 6B).

### LK-A exhibits anti-tumour activity in CNE2 xenograft tumour models

**Figure 7:**
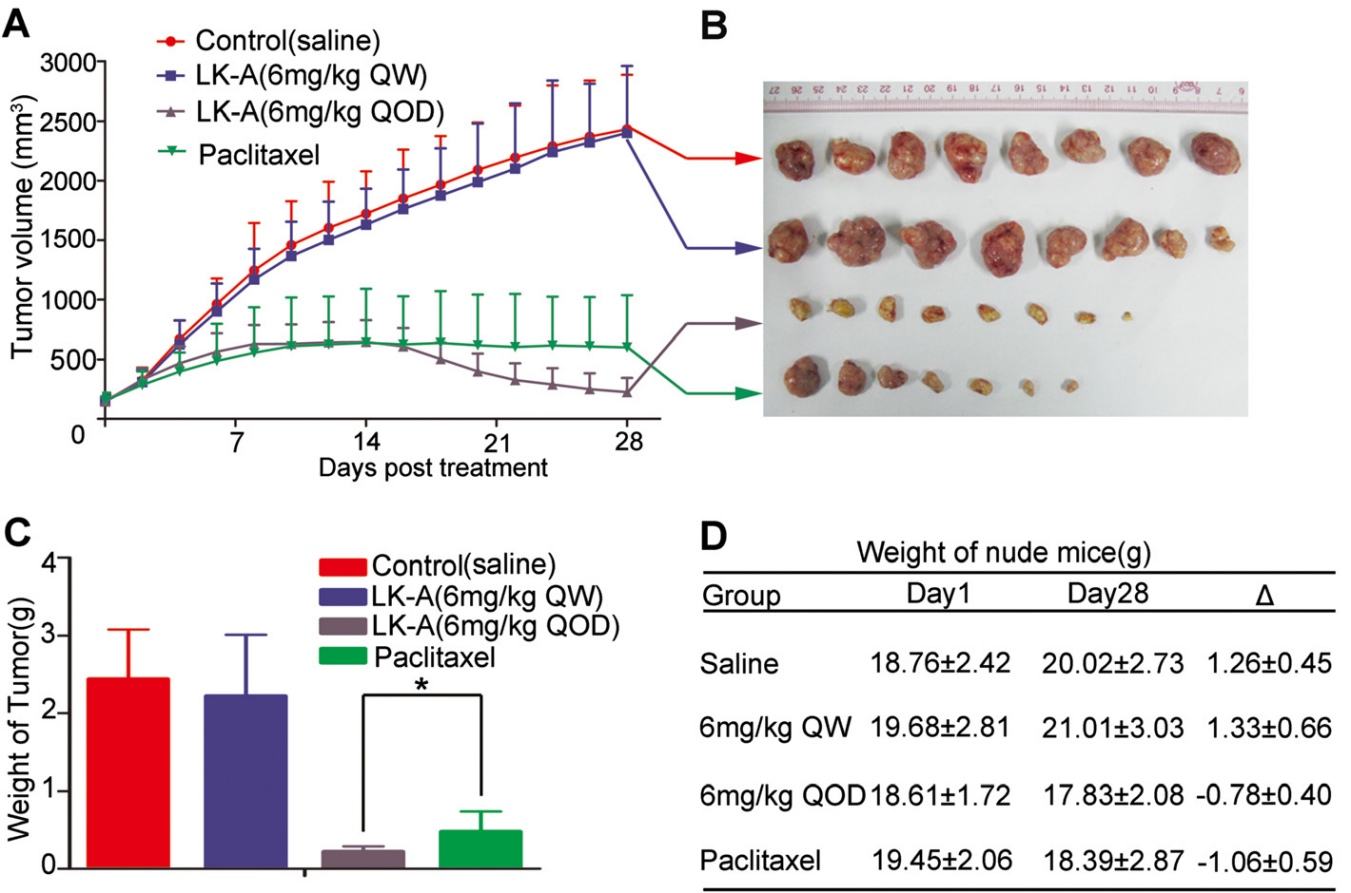
**LK-A exhibits anti-tumour activity *in vivo*.** Mice bearing CNE2 nasopharyngeal carcinoma tumours were treated with LK-A (6 mg/Kg QW or 6 mg/Kg QOD), Paclitaxel or saline for three weeks. **(A, B)** Graph and images depicted the tumour volumes (mm^3^) over time (data are shown as the mean tumour volume ± SD). **(C)** Bar chart shows the weight of tumours from mice (data are shown as the mean tumour weight ± SEM *p < 0.05). **(D)** The graph shows the weight change of the nude mice. On first day there are no differences between the four groups. On the 28th day, there is a significant change in body weight in mice treated with LK-A (6 mg/Kg QOD) or Paclitaxel compared with mice treated with LK-A (6 mg/Kg QW) or the saline group (*p < 0.05). However, there is no difference between the LK-A-treated (6 mg/Kg QOD) and Paclitaxel-treated groups.

Finally, we examined whether LK-A exhibits anti-tumour activity in xenograft models. Paclitaxel is widely used in the treatment of NPC patients. Previous studies have shown that paclitaxel can exert significant cytotoxic effects on NPC cells just as described in Additional file 1: Figure S1C. So we choosed Paclitaxel-treated mice as positive control group. As shown in Figure 7A, 7B and 7C, the tumour growth rate and tumour weight of tumours from mice treated with a high-dose of LK-A were much lower than Paclitaxel-treated mice (positive control group) (*P < 0.05). However, there was no difference in the tumour growth rate and tumour weight for tumours from mice treated with low-dose LK-A and mice treated with saline. There was no significant reduction in the weights of the mice treated with either LK-A or Paclitaxel (Figure 7D). Collectively, the potential anti-tumour effect of LK-A was equivalent to that of Paclitaxel and virtually no acute toxicity, at least in the weights of the mice, was observed.

## Discussion

Natural agents with anti-cancer effects will certainly play an important role in the development of new anti-tumour drugs [23]. Because of their unique carbon skeleton and multiple pharmacological properties, diterpenes have recently gained much attention as potential candidates for new treatments against cancer.

LK-A is a natural *ent*-kaurene diterpenoid isolated from *Isodon* genus. To date, only one study has been performed on the anti-cancer effects of LK-A, and it demonstrated that LK-A has cytotoxic activity on multiple myeloma H929 cells [9]. In the present study, LK-A exhibited higher anti-tumour activity on NPC cell lines than oridonin. At concentrations that were much lower than the IC_50_ value, LK-A still exhibited significant anti-tumour activity during long-term treatment. LK-A inhibited cell growth of NPC cells by inducing apoptosis through the intrinsic caspase pathway and causing cell cycle arrest. *In vivo*, LK-A exhibited anti-tumour activity comparable to Paclitaxel.

Many previous studies have already confirmed that *ent*-kaurane diterpenoids, such as oridonin and eriocalyxin B, which contains an *α*,*β*-unsaturated ketone group, have significant anti-tumour activity [24]. In the present study, LK-A exhibited more potent anti-tumour activity on NPC cell lines than oridonin. This may be because a hydroxyl group at the C-1 position of LK-A is absent compared with oridonin (Figure 1A). This phenomenon is very common in *ent*-kaurane compounds [25-27]. Similar to other *ent*-kaurane diterpenoids, LK-A inhibited cell growth of NPC cells by inducing apoptosis and causing cell cycle arrest. However, as far as we know, no specific target has been identified for any *ent*-kaurane diterpenoids that may explain these effects.

The anti-tumour activity of *ent*-kaurane diterpenoids may be due to their ability to induce cancer cell apoptosis. The P13K/Akt signalling pathway plays an important role in cell proliferation and survival. It has been shown that oridonin may suppress constitutively activated targets of phosphatidylinositol 3-kinase (including Akt, FOXO, and GSK3) in HeLa cells, which inhibits their proliferation and the induction of caspase-dependent apoptosis [28]. In agreement with these data, the expression levels of phospho-Akt and phospho-Gsk-3β were decreased in NPC cells treated by LK-A (Figure 5B). This change may alter the expression of Bcl-2 family proteins [29]. Proteins of the Bcl-2 family either promote cell survival (Bcl-2 and Bcl-x_L_) or induce programmed cell death (Bax). The ratio of Bax/Bcl-2 is critical for determining whether apoptosis will be induced [30]. We found that treatment of NPC cells with LK-A resulted in an increase in the expression of Bax and the ratio of Bax to Bcl-x_L_ (Figure 5A). This increase may cause a loss of mitochondrial membrane potential and a consequent release of cytochrome c from mitochondria into the cytosol. Release of cytochrome c activates the caspase cascade and PARP cleavage to execute the apoptotic program [31-33]. Our data showed that treatment of cells with LK-A caused a dose-dependent activation of caspase-9, caspase-3 and PARP (Figure 3), while a pan-caspase inhibitor (Z-VAD-FMK) attenuated this LK-A-induced apoptosis (Figure 4A). Our results suggest that LK-A induces caspase-dependent apoptosis in NPC cells.

Cell cycle progression is a hallmark for cell proliferation. Deregulation of the cell cycle has been linked with cancer initiation and progression [34]. Control of cell cycle progression in cancer cells is considered to be a potentially effective strategy for the control of tumour growth [35,36]. NPC cells treated with 0.1 or 0.2 μM LK-A had a higher proportion of cells in higher S phase and fewer cells in G2/M phase compared with untreated cells (Figure 6). These data indicate that LK-A may induce S phase arrest. However, further work is necessary to determine the detailed mechanism of LK-A-induced cell cycle arrest in NPC cells.

Many diterpenoids have been proven to have significant anti-tumour effects *in vivo*[8,37] (Fan QQ et al., 2010; Zhou GB et al., 2007). *In vivo*, LK-A administered every other day exhibited a similar or even better inhibitory effect on CNE2 cell proliferation than Paclitaxel did. Furthermore, there was virtually no acute toxicity observed; this finding suggests that LK-A is a relatively safe agent (Figure 7). However, there was no anti-tumour effect when LK-A was given once a week. This may be due to a short half-life of LK-A in nude mice, although further investigation is required to empirically test this hypothesis. These data suggests that LK-A could be effective in treating human nasopharyngeal carcinoma.

## Conclusion

We first showed that LK-A inhibited NPC cell proliferation through the induction of cell cycle arrest and apoptosis. At low concentrations far less than the IC_50_ values, LK-A primarily arrested NPC cells in S phase. More importantly, at these low concentrations, LK-A significantly inhibited the colony formation of the NPC cells. Therefore, it will be interesting to determine whether low doses of LK-A plus radiotherapy or chemotherapy drugs exhibit a significant synergistic effect on the treatment of nasopharyngeal carcinoma without increasing toxic side effects. *In vivo,* the anti-tumour effects of LK-A were comparable with those of Paclitaxel, indicating that LK-A may be developed as a new specific and attractive therapy for NPC.

## Endnotes

In Figure 6 section A: the number of all horizontal axises is 0, 32, 64, 96, 128, 160, 192, 224, 256; the number of vertical axis in the first photo (CNE1 control) is 0, 110, 220, 330, 440, 550, 660; the number of vertical axis in the second and third photo (CNE1 0.1 μM, CNE1 0.2 μM) is 0, 70, 140, 210, 280, 350, 420; the number of vertical axis in the fourth photo (CNE2 control) is 0, 80, 160, 240, 320, 400, 480; the number of vertical axis in the fifth photo (CNE2 0.1 μM) is 0, 150, 300, 450, 600, 750, 900; he number of vertical axis in the sixth photo (CNE2 0.2 μM) is 0, 140, 280, 420, 560, 700, 840.

## Competing interests

The authors declare that they have no competing interests.

## Authors’ contributions

QFZ and JKD carried out the experimental studies, drafted the graphs, performed the statistical analysis and wrote the paper. HZ, HBW and YD have been involved in the experimental technical support. SPC and DX have been involved in revising figures. ZDH and MZL have been involved in the animal experiment. JZ, HDS and JXP isolated longikaurin A from *Isodon* genus. MSZ and JXP have been involved in the design of the study and revising critically the manuscript and have given final approval of the version to be published. All authors read and approved the final manuscript.

## Supplementary Material

Additional file 1: Figure S1 Proliferation assay by MTT assay. **(A)** A comparison of the cytotoxic effects of LK-A and oridonin on immortalised nasopharyngeal epithelial cells (NPEC2-Bmi-1). The IC_50_ values at 48 hrs after treatment with LK-A and oridonin were 2.96 ± 0.32 μM and 3.15 ± 0.48 μM NPEC2-Bmi-1 cells, respectively. Data are shown as the mean ± SD of three independent experiments. **(B)** Curves chart showing the inhibition of cell viability for LK-A and oridonin accordingly for 48 hrs and 72 hrs with CNE-1, -2 and NEPC2-Bmi1. Cell viability was determined by MTT assays. **(C)** CNE1, CNE2 and NEPC2-Bmi1 (0, 0.5, 1, 2, 4, 8 and 16 μΜ) of Paclitaxel for 24, 48 and 72 hrs. Cell viability was determined by MTT assays. The IC_50_ values 48 hrs after treatment were 1.8 ± 0.14 μM, 2.35 ± 0.17 μM and 3.16 ± 0.27 μM for CNE1, CNE2 and NEPC2-Bmi1 cells, respectively. Cell viability was determined by MTT assays. Data are shown as the mean ± SD of three independent experiments. Click here for file

Additional file 2: Figure S2 Flow cytometry analysis of NEPC2-Bmi1cells treated with 0.78, 1.56 and 3.12 μΜ LK-A for 48 hrs. **(A)** Dot plots showing the percentage of viable (D3), early apoptotic (D4), late apoptotic (D2) and necrotic (D1) cells. **(B)** Bar chart indicating the increased proportion of early and late apoptotic cells after treatment with LK-A. Data are shown as the mean ± SD from two independent experiments. Click here for file
