# Supplementary figures and images for: Anti-tumour activity of longikaurin A (LK-A), a novel natural diterpenoid, in nasopharyngeal carcinoma
Source: J Transl Med. 2013 Aug 28;11:200. doi: 10.1186/1479-5876-11-200 (PMC3847153; doi:10.1186/1479-5876-11-200)

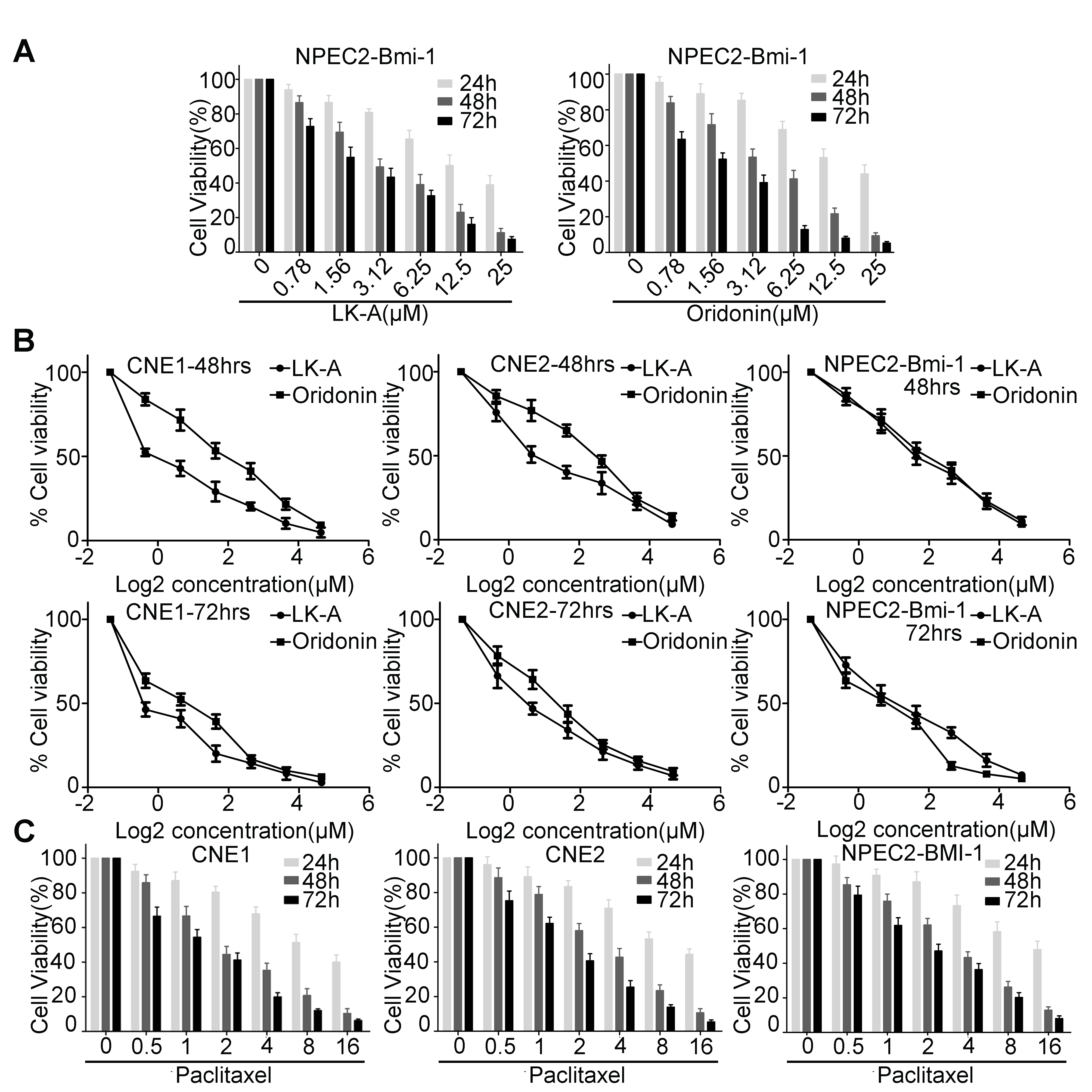

Supplement: Additional file 1: Figure S1 — Proliferation assay by MTT assay. (A) A comparison of the cytotoxic effects of LK-A and oridonin on immortalised nasopharyngeal epithelial cells (NPEC2-Bmi-1). The IC50 values at 48 hrs after treatment with LK-A and oridonin were 2.96 ± 0.32 μM and 3.15 ± 0.48 μM NPEC2-Bmi-1 cells, respectively. Data are shown as the mean ± SD of three independent experiments. (B) Curves chart showing the inhibition of cell viability for LK-A and oridonin accordingly for 48 hrs and 72 hrs with CNE-1, -2 and NEPC2-Bmi1. Cell viability was determined by MTT assays. (C) CNE1, CNE2 and NEPC2-Bmi1 (0, 0.5, 1, 2, 4, 8 and 16 μΜ) of Paclitaxel for 24, 48 and 72 hrs. Cell viability was determined by MTT assays. The IC50 values 48 hrs after treatment were 1.8 ± 0.14 μM, 2.35 ± 0.17 μM and 3.16 ± 0.27 μM for CNE1, CNE2 and NEPC2-Bmi1 cells, respectively. Cell viability was determined by MTT assays. Data are shown as the mean ± SD of three independent experiments. [file 1479-5876-11-200-S1.tif]

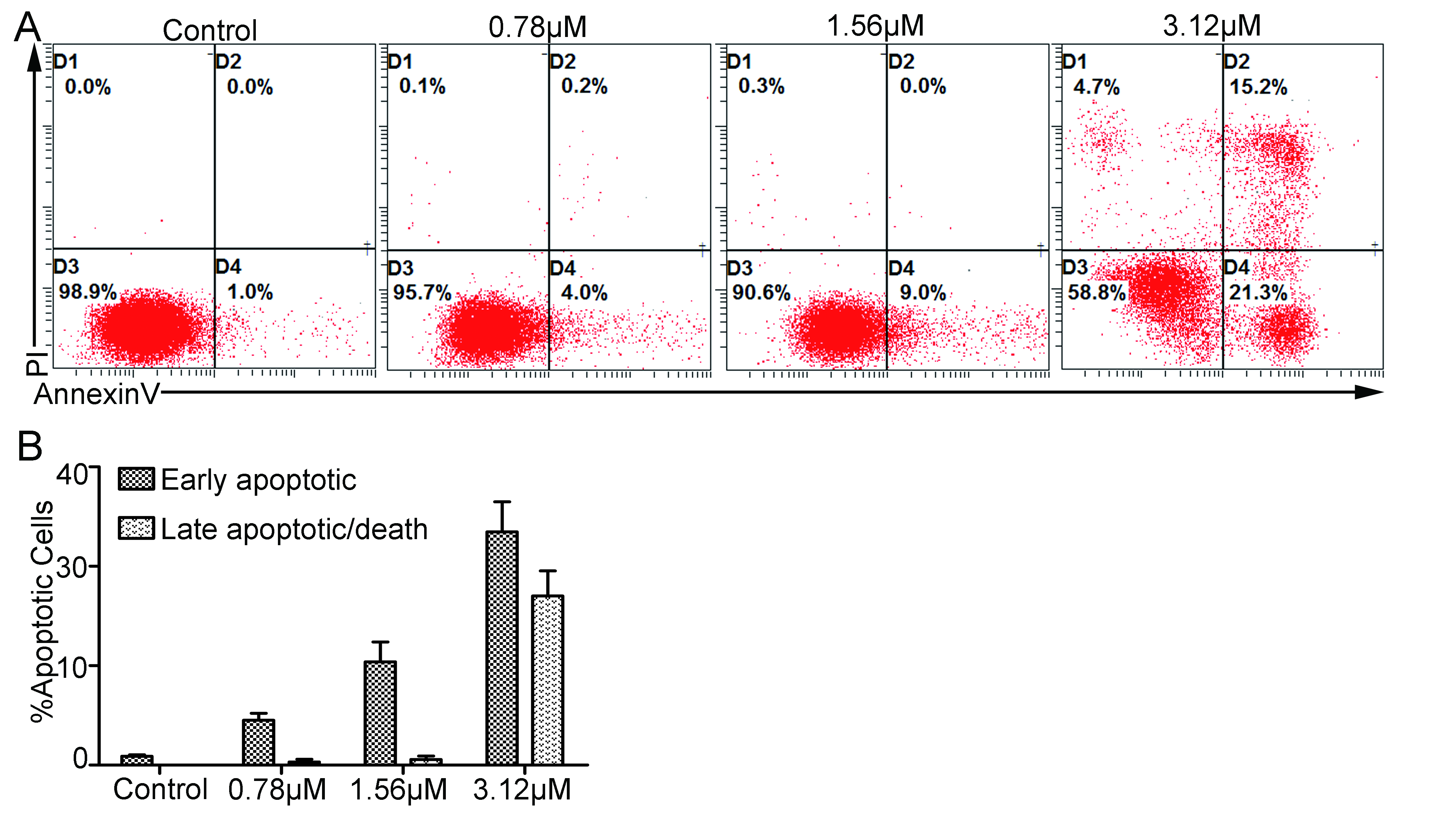

Supplement: Additional file 2: Figure S2 — Flow cytometry analysis of NEPC2-Bmi1cells treated with 0.78, 1.56 and 3.12 μΜ LK-A for 48 hrs. (A) Dot plots showing the percentage of viable (D3), early apoptotic (D4), late apoptotic (D2) and necrotic (D1) cells. (B) Bar chart indicating the increased proportion of early and late apoptotic cells after treatment with LK-A. Data are shown as the mean ± SD from two independent experiments. [file 1479-5876-11-200-S2.tif]
